# Landscape Effects on the Abundance of *Apolygus lucorum* in Cotton Fields

**DOI:** 10.3390/insects11030185

**Published:** 2020-03-14

**Authors:** Minlong Li, Long Yang, Yunfei Pan, Qian Zhang, Haibin Yuan, Yanhui Lu

**Affiliations:** 1 College of Agronomy, Jilin Agricultural University, Changchun 130118, China; li694449602@163.com (M.L.); Yhb-74@163.com (H.Y.); 2 State Key Laboratory for Biology of Plant Diseases and Insect Pests, Institute of Plant Protection, Chinese Academy of Agricultural Science, Beijing 100193, China; yanglong9005@163.com (L.Y.); yunfeipanpan@126.com (Y.P.); zhangqian9405@163.com (Q.Z.)

**Keywords:** Miridae, polyphagous pests, agricultural landscape, resources continuity, population outbreak, regional pest management

## Abstract

Resource-continuity over spatial and temporal scales plays a central role in the population abundance of polyphagous pests in an agricultural landscape. Shifts in the agricultural land use in a region may alter the configuration of key resource habitats, resulting in drastic changes in pest abundance. *Apolygus lucorum* (Meyer-Dür) (Hemiptera: Miridae) is a pest of cotton in northern China that has become more serious in recent years following changes in the region’s cropping systems. However, no evidence from the landscape perspective has yet been gathered to account for the increasing population of *A. lucorum* in China. In this study, we investigated the effects of landscape composition on the population abundance of *A. lucorum* in cotton fields in July and August of 2016, respectively. We found that increased acreage planted to cotton actually had a negative effect on the abundance of *A. lucorum*, while planting of other crops (e.g., vegetables, soybean, and peanut) was positively associated with the mirid’s population abundance in cotton fields. Maize production only displayed a positive effect on population abundance in August. Our results suggested that the decreasing of cotton area may weaken the trap-kill effect on *A. lucorum*, and the extension of other crops and maize potentially enhance the continuity of resources needed by *A. lucorum*. Combined effects of these two aspects may promote an increased population density of *A. lucorum* in the agriculture district. In the future, when possible, management strategies in key regional crops should be coordinated to reduce resource continuity at the landscape or area-wide scale to lower *A. lucorum* populations across multiple crops.

## 1. Introduction

Promoting ecosystem services (such as pest suppression, pollination, and nutrient cycling) on a landscape or area-wide scale to lessen the need for agricultural inputs is crucial for achieving sustainable agriculture [1,2,3]. In agricultural landscapes, habitats especially non-crop habitats that suffer less extensive disturbance, provide pests and insect natural enemies with necessary resources, food and shelter to complete their life cycles throughout the year [4,5,6]. However, changes to the cropping pattern and cropland expansion have altered the spatial and temporal configuration of these habitat resources, usually resulting in drastic changes in the occurrence of agricultural insects [7,8,9,10,11]. Identifying the critical landscape habitats that contribute to pest outbreaks then becomes an important part of sustainable agricultural production.

For polyphagous herbivores, spatial and temporal resource continuity is one of the main prerequisites of a thriving population [12,13,14,15]. Agricultural landscapes characterized by continuity of the resources needed by a pest over an entire season are usually associated with greater pest pressure [9,16,17]. In contrast, resource discontinuity caused by limited or low quality of available resources will result in a reduced pest population [7,18]. Investigating population dynamics over time at a landscape level is a promising approach for identifying the source and sink habitats of pests in a particular period [13]. Combined with information on the biology of a particular pest, we can determine the roles played by different habitats in resource continuity and estimate the relative importance of different habitats during a specific time [14]. Understanding the ecological mechanisms involved in the changing status of a crop pest as cropping systems evolve, will allow for more targeted strategies to interrupt resource continuity and suppress pest density.

In northern China, *Apolygus lucorum* (Meyer-Dür) (Hemiptera: Miridae) makes use of more than 200 plant species [19,20]. Adults have high dispersal ability and can cause serious damage to cotton and a number of fruits and vegetables. They seek out and lay eggs on flowering plants because flowers are a key feeding site for both nymphs and adults [19,20,21,22,23]. *Apolygus lucorum* has five generations per year in this region [24], the first two of which mainly feed on fruit trees and weedy plants from April to May; in mid-to-late June adults of the second generation migrate into cotton, third and fourth generations feed on cotton and some other crops from July to August; finally, adults of the fifth generation return to fruit trees and weeds to overwinter [19,24,25]. From the late 1990s to the early 2000s, Bt (*Bacillus thuringiensis*) cotton adoption effectively suppressed cotton bollworm [*Helicoverpa armigera* (Hübner)], resulting in a reduction of insecticide sprays applied in cotton fields and a subsequent outbreak of mirid bugs (including *A. lucorum*) in many crops [9]. After 2010, however, the area planted to cotton decreased sharply, with the expansion of maize and some other crops such as vegetables, soybeans, and peanut. *Apolygus lucorum* experiences little pressure from natural enemies [26,27], so large increases in the availability of host plants might have a pronounced effect on *A. lucorum* densities in this region. However, the influence of land use changes on *A. lucorum* population abundance in cotton fields has rarely been studied.

In this study, we investigated the effects of landscape composition on the abundance of *A. lucorum* in cotton fields in July and August, respectively. Specifically, we surveyed *A. lucorum* density using sex pheromone traps in these two periods, and used the regression approach to model the mirid’s abundance as a function of landscape variables. Our study aims to answer the following questions: (1) Do landscapes characterized by a high proportion of cotton negatively affect the abundance of *A. lucorum* in cotton fields?; (2) does a high proportion of other crops and maize increase the abundance of *A. lucorum* in cotton fields?; and (3) do relationships between landscape variables and *A. lucorum* population abundance change in different months?

## 2. Materials and Methods

### 2.1. Study Locations

**Figure 1:**
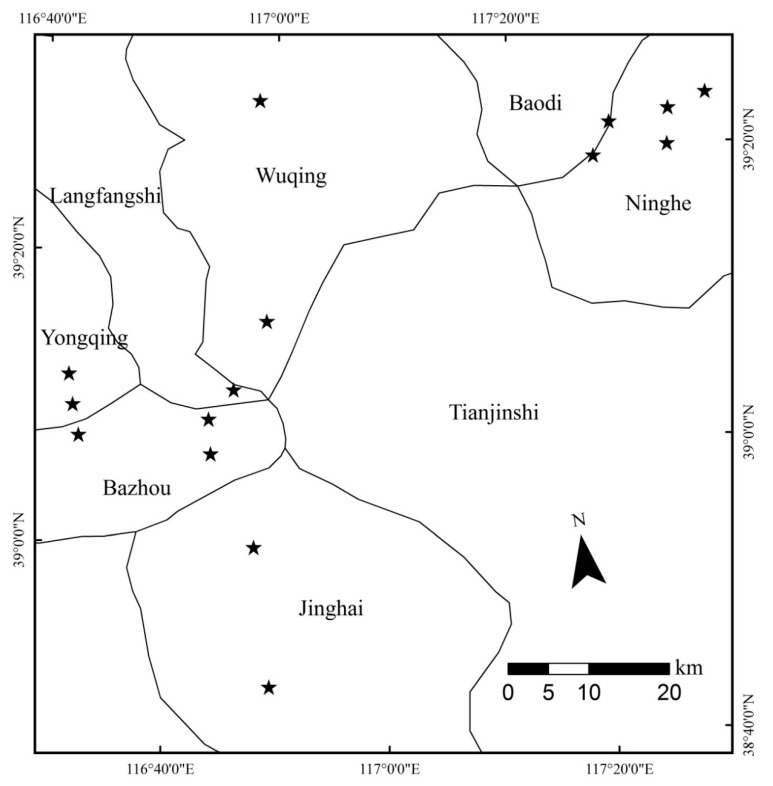
Location of 15 cotton fields in Langfang (Langfangshi, Yongqing and Bazhou) and Tianjin (Baodi, Ninghe and Jinghai) regions.

The survey was conducted in 15 commercial cotton fields in Langfang (Langfangshi, Yongqing, and Bazhou) and Tianjin (Baodi, Ninghe, and Jinghai) cities in northern China (Figure 1) in 2016. The study region was one of the major cotton-producing areas in China several years ago, but cotton acreage has declined significantly in recent years. Study sites were selected after a preliminary screening through Google Earth, the selected fields were located within landscapes with a gradient of proportions for cotton, maize, and other crops. The cotton fields sampled averaged 6.19 ± 1.58 ha (mean ± SEM).

### 2.2. Sampling Method

The abundance of male *A. lucorum* captured in pheromone-baited traps was used as a proxy for estimating *A. lucorum* density, as pheromone trapping is an appropriate method to monitor its population [28]. Bucket traps (Pherobio Technology Co., Ltd., Beijing, China) baited with a commercial pheromone lure (Pherobio Technology Co., Ltd., Beijing, China) were used for trapping. The primary components of the sex pheromone were 4-oxo-(*E*)-2-hexenal and trans-2-hexenyl butyrate [28,29]. We added some water and detergent to each bucket to prevent the escape of *A. lucorum* adults. Previous studies have found that *A. lucorum* damage cotton most seriously in July and August, as cotton is highly vulnerable during this period [30], therefore in this study we focused on these two months. The trapping was conducted in mid-to-late July (peak period for the third generation) and in mid-to-late August (peak period for the fourth generation). Five bucket traps (situated about 15 cm above the top of the cotton) were randomly arranged in each cotton field, at least 15 m apart. Each month two sampling rounds were conducted in two consecutive weeks, and each sampling round lasted three days. The number of *A. lucorum* in each bucket was counted weekly, at each time adults were removed and lures replaced. Data for the two trapping events for each month were averaged.

### 2.3. Land Use Data and Analysis

Following the approach used by Yang et al. [31], we calculated the proportion of each habitat surrounding each cotton field, working outward from the center of each cotton field 0.5, 1.0, 1.5, and 2.0 km, using ArcGIS 10.2 software [32]. For each spatial scale, we measured the percentage of the total area covered by each of six landscape types: cotton, dwellings (roads and dwellings), maize, other crops (vegetables, greenhouse, fruit trees, soybeans and peanuts), woodlots (poplar trees and reforested areas), and bodies of water (Figure S1). A Spearman correlation test was performed to check for correlations among landscape variables (% of land in six categories) (Table S1). Water area displayed a strong correlation with other crops at each scale, and water bodies were assumed to serve no biological function for *A. lucorum*, so we excluded water from future analyses. The variance inflation factor (VIF) was calculated for the remaining predictors at each spatial scale, and VIF values were found to be below 3.0, indicating that covariation in the remaining five landscape variables was not a problem [33].

### 2.4. Statistical Analysis

Linear models were used to investigate the relationships between landscape variables and the abundance of *A. lucorum* trapped in cotton fields in July and August. Before fitting data to models, the trap catch of *A. lucorum* was log10(x + 1) transformed, and landscape variables (% of land in five of our six categories: other crops, cotton, maize, woodlots, dwellings) were logit transformed and standardized for model convergence and normality. In all models, the explanatory variables were considered in an additive way only, and there are 32 alternative candidate models at each scale.

Bias-corrected Akaike’s information criterion (AICc), which corrects for small sample size, was used to compare and rank all alternative candidate models at each spatial scale [34], after which a model averaging procedure was used to reduce model selection bias and obtain robust results [35,36]. To avoid redundant models and spurious results, we used the top 4 AICc values to define the top model set for model averaging (i.e., models with a ΔAICc less than 4 were used in model averaging), and derived coefficients for variables from the top model set [36]. The model selection progress revealed the relative importance of explanatory variables and relationships between the response and explanatory factors. Relative importance values for variables were quantified by the sum of the Akaike weights associated with each variable in models in the top model set [36].

Models were systematically assessed by examining the model normality and homoscedasticity of the residuals. We also conducted Moran’s test for the residuals of all models to test for spatial autocorrelation and found no evidence of autocorrelation. All analyses were conducted in R, using the “stats” package for linear models [37], the “MuMIn” package for model averaging [38], and the “spdep” package for Moran’s test [39].

## 3. Results

### 3.1. Abundance of A. lucorum

We counted a total of 12248 *A. lucorum* individuals during the sampling. The abundance of *A. lucorum* adults in July was 36.30 ± 5.15 males per trap per week (Mean ± SEM), and was 44.12 ± 6.64 per trap for the August.

### 3.2. Landscape Effects on Abundance of A. lucorum in July

**Figure 2:**
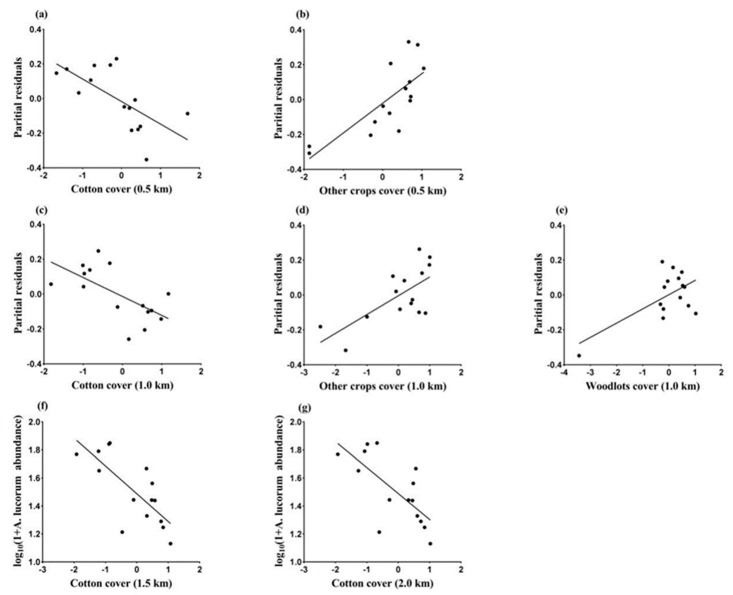
Relationships between the abundance of *Apolygus lucorum* in July and landscape variables with the best fitting model at each scale (0.5 km: (**a**,**b**); 1.0 km: (**c**–**e**); 1.5 km: (**f**); 2.0 km: (**g**)), based on AICc analysis. The proportion of different landscape types cover was logit transformed and standardized.

The model with cotton and other crops had the lowest AICc value at the 0.5 km spatial scale, which suggest that a high proportion of other crops within the landscape benefited *A. lucorum* abundance and the area of cotton had a negative effect (Figure 2a,b). Meanwhile, the model with cotton, other crops, and woodlots had the lowest AICc value at a 1.0 km radius, indicating that other crops within this scale increased the abundance of *A. lucorum* trapped in cotton fields, and cotton planting was not beneficial for the abundance of *A. lucorum* (Figure 2c–e). The model with cotton alone was the best at the larger spatial scales (1.5 and 2.0 km) (Table 1), the proportion of cotton at these two scales weaken the occurrence of *A. lucorum* in cotton fields (Figure 2f,g). AICc values for models at the smaller scales (0.5 and 1.0 km) were much lower than those at larger scales (1.5 and 2.0 km), suggesting that the smaller scales are more suitable for predicting the abundance of *A. lucorum* adults in cotton fields in relation to landscape variables during this period. Across the four scales, the variance explained by the best model at each scale ranged from 45 to 67% (Table 1).

Model averaging at the 0.5 km scale showed that cotton and other crops within the landscape had the strongest negative and positive effects, respectively, on the population abundance of *A. lucorum*, given that the averaged estimated coefficients’ absolute values and their relative importance values were the highest. At the 1.0 km scale, model averaging procedure suggested that other crops and woodlots had the strongest positive effects on *A. lucorum*, while cotton area had the strongest negative effects. At the 1.5 and 2.0 km scales only cotton had significantly negative effects on *A. lucorum*. Other landscape variables such as maize and dwellings displayed no significant effects on the abundance of *A. lucorum* (Table 2).

### 3.3. Landscape Effects on Abundance of A. lucorum in August

**Figure 3:**
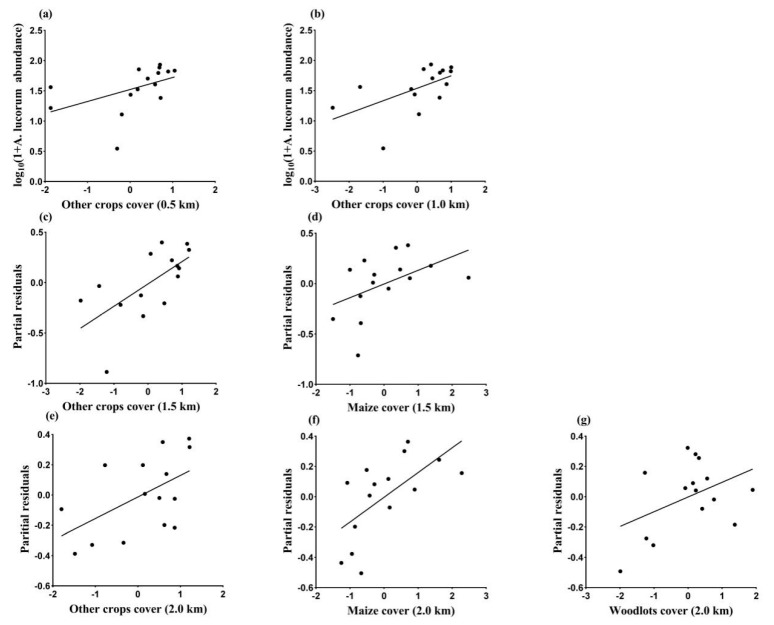
Relationships between the abundance of *Apolygus lucorum* in August and landscape variables with the best fitting model at each scale (0.5 km: (**a**); 1.0 km: (**b**); 1.5 km: (**c**,**d**); 2.0 km: (**e**–**g**)), based on AICc analysis. The proportion of different landscape types cover was logit transformed and standardized.

Abundance of *A. lucorum* in August was best described by the model with other crops, which was the best fitting model with the lowest AICc value, at the 0.5 and 1.0 km spatial scales. In these smaller scales, other crops displayed a positive effect on *A. lucorum* abundance (Figure 3a,b). The model with maize and other crops was the best at the 1.5 km radius, partial residual plot of the model suggested that both maize and other crops areas within landscape are beneficial for *A. lucorum* (Figure 3c,d). Across the four studied scales, the model with maize, other crops, and woodlots at the 2.0 km scale had the lowest AICc value, indicating that 2.0 km was the best scale for estimating the effects of landscape composition on *A. lucorum*. In this largest scale maize, other crops and woodlots benefited the occurrence of *A. lucorum* (Figure 3e–g). The variance explained by the best model at each scale ranged from 17 to 64% (Table 3).

The results of model averaging procedures showed that the proportion of other crops had the strongest positive effect on the abundance of *A. lucorum*, as this landscape factor had the highest averaged estimated coefficient and relative importance values across four spatial scales. Meanwhile, maize area within 1.5 and 2.0 km also showed a positive correlation with *A. lucorum* abundance during this period (Table 4).

## 4. Discussion

In an agricultural landscape, the spatial and temporal configuration of food resources play a pivotal role in the population dynamics of polyphagous pests [13,40,41]. Here, we found that crop habitats such as cotton, maize, and other crops (vegetables, soybean, peanut, etc.,) had variable relationships with the abundance of *A. lucorum* in cotton fields. These crop habitats might play different roles, which change over time, in the continuity of resources for *A. lucorum*. As expected, cotton area had a negative effect on the abundance of *A. lucorum* in July. Meanwhile the presence of other crops raised the abundance of *A. lucorum*. Maize production only had a positive effect on the abundance of *A. lucorum* in the later period. Combining these cultivated crops as a single landscape variable, our previous country-level analysis found that land use diversity (Shannon diversity) negatively affected mirid bug severity; the proportions of forest, water bodies, and unused land area were all negatively correlated with pest severity [42]. Here, we provided a more disaggregated cultivated land use classification, and estimated their effects separately on the population abundance of *A. lucorum*, revealing the functions of different host crops within a landscape more explicitly.

The lower abundance of third generation *A. lucorum* adults in landscapes with more cotton, may be due to a dilution effect caused by the large cotton area. Cotton is one of the few flowering host plants available to *A. lucorum* in July, and adults of *A. lucorum* tend to migrate from other host plants into flowering cotton fields, and consequently larger areas of cotton may result in a lower density of *A. lucorum* per unit area. This dilution effect becomes weaker in August because more and more host crops start to flower. 

In addition, insecticide use frequently in cotton fields may also contribute to the responses of *A. lucorum* to the proportion of cotton area in the landscape. July is one of the main periods of cotton vulnerability to aphids, cotton bollworm and *A. lucorum*, and broad-spectrum insecticides are commonly used in cotton fields during this time [43]. During this period, flowering cotton attracts *A. lucorum* into cotton fields and intensive insecticide application kills them, such that cotton works as a dead-end trap crop, and cotton therefore suppresses the abundance of *A. lucorum* in the broader agricultural landscape [9,19]. Carrière et al. [13] also found a consistent negative association between the density of mirid bugs in cotton fields and cotton area within a landscape, owing to the use of insecticides during the fruiting period.

Maize area was positively associated with the abundance of the fourth generation of *A. lucorum*, but it appeared to have no effect on the third generation. Mirid bugs can only feed on corn ears and tassel under field conditions [44,45], and this may make maize fields a suitable habitat for *A. lucorum* at the landscape level in a particular period. August coincides with the silking stage for maize in our study region, maize fields can provide alternative food resources for *A. lucorum* and increase population abundance in cotton fields. Furthermore, the relative physical height of maize leads to lower insecticide intensity. As a result, maize fields may serve as a refuge habitat for *A. lucorum* while cotton fields are sprayed with insecticides during this period. In contrast, lower food resources in maize fields during earlier period could explain the lack of any significant relationship between maize and the abundance of *A. lucorum* in the earlier period. Through stable carbon isotope signatures analysis, Jiao [46] also found that the proportion of *A. lucorum* adults that developed on C_4_ plants for the fourth generation was much higher than for the third generation in northern China. In summary, the food resource provided by maize in appropriate growth stage may promote the abundance of fourth generation *A. lucorum* in cotton fields.

The abundance of crop habitats other than cotton and maize also enhanced *A. lucorum* numbers in July and August, indicating that crops like vegetables, fruits, and beans were also source habitats from which *A. lucorum* migrates into cotton fields during this period. The long period of time with inflorescences and tender plant tissues of these other crops makes them a suitable food resource for *A. lucorum* [24,30], contributing to the mirid bug’s abundance in cotton fields. Under field conditions, many host plants, such as *Vigna radiata*, *Helianthus annuus*, and *Ricinus communis*, usually sustain high-level population of *A. lucorum* in summer season [20,47,48]. Other crops in a landscape may also make it convenient for *A. lucorum* adults to switch host plants, further promoting their population growth [49]. Furthermore, in Chinese small-holder plantings, these other crops usually belong to different farmers, and planting and management schedules also differ among fields. Lacking a coordinated and uniform set of agricultural practices, these crops, can increase resource continuity for *A. lucorum* and provide more pest sources for cotton fields.

In north China, widespread adoption of Bt cotton resulted in a cycle where reduced need for insecticides promoted biological services, further decreasing insecticide use in cotton fields [10], resulting in an outbreak of mirid bugs in various other crops [9]. In recent years, the reduction in cotton production has again weakened this dead-end trap effect, because the number of insecticide sprays used in Bt cotton is still much higher than in other crops. The expansion of other crops and maize, which are less frequently exposed to insecticides, could provide more suitable host plants for this pest. Both of these factors account at least in part for the increased damage in crops from *A. lucorum* recently in northern China.

## 5. Conclusions

In summary, the results of our study, although performed for one year only, highlight the potential role of decreased cotton area, and the extension of other crops (including maize), on the increased density of *A. lucorum*. The amounts of suitable habitats in an agricultural landscape promote resource continuity for *A. lucorum* in both space and time, resulting in an increased population density in northern China. Management actions should be implemented on targeted host crops to interrupt resource continuity and lower *A. lucorum* populations at the landscape or area-wide scale. In particular, smallholder agricultural systems would benefit from coordinated and uniform management strategies in the key host habitats for the control of *A. lucorum*.

## Figures and Tables

**Table 1:** List of models that were included in the top model set (ΔAICc < 4.0) for model averaging procedure, to infer landscape effects on the abundance of *Apolygus lucorum* in cotton fields in July. Best models at each scale are indicated in bold.

Scales (km)	Model	K	logLik	AICc	ΔAICc	Weight	Adjusted *R*^2^
0.5	Cotton + Other crops	4	10.00	−7.99	0.00	0.43	0.67
	Cotton + Other crops + Woodlots	5	12.17	−7.66	0.33	0.36	0.73
	Cotton + Dwellings + Other crops	5	10.72	−4.76	3.23	0.08	0.67
	Cotton + Dwellings + Other crops + Woodlots	6	13.38	−4.26	3.73	0.07	0.75
	Cotton + Maize + Other crops	5	10.37	−4.08	3.92	0.06	0.66
1.0	Cotton + Other crops + Woodlots	5	12.57	−8.47	0.00	0.46	0.74
	Cotton + Maize + Other crops + Woodlots	6	14.97	−7.45	1.02	0.28	0.79
	Cotton + Other crops	4	9.02	−6.04	2.42	0.14	0.62
	Maize + Other crops + Woodlots	5	11.2	−5.74	2.72	0.12	0.74
1.5	Cotton	3	6.74	−5.30	0.00	0.33	0.53
	Cotton + Woodlots	4	8.46	−4.93	0.37	0.27	0.59
	Cotton + Other crops	4	7.82	−3.65	1.65	0.14	0.56
	Cotton + Dwellings	4	7.52	−3.04	2.26	0.11	0.54
	Cotton + Other crops + Woodlots	5	9.24	−1.80	3.50	0.06	0.60
	Cotton + Maize	4	6.83	−1.67	3.63	0.05	0.49
	Other crops	3	4.75	−1.33	3.97	0.04	0.38
2.0	Cotton	3	5.61	−3.04	0.00	0.44	0.45
	Cotton + Woodlots	4	6.5	−1.00	2.04	0.16	0.47
	Cotton + Other crops	4	6.24	−0.49	2.55	0.12	0.45
	Cotton + Dwellings	4	5.88	0.25	3.29	0.08	0.43
	Woodlots	3	3.83	0.52	3.56	0.07	0.30
	Cotton + Maize	4	5.61	0.78	3.82	0.06	0.40
	Other crops	3	3.7	0.79	3.82	0.06	0.29

**Table 2:** Results of model averaging procedure to estimate landscape effects on abundance of *Apolygus lucorum* in cotton fields in July. Significant at: ^*^ *p* < 0.05; ^**^ *p* < 0.01; ^***^ *p* < 0.001.

Scales (km)	Variable	Estimate	z Value	Pr (>|z|)	Relative Importance
0.5	Intercept	1.511	39.271	< 0.001 ^***^	
	Cotton	−0.222	2.676	0.007 ^**^	1.00
	Dwellings	−0.085	1.027	0.304	0.15
	Maize	−0.067	0.669	0.503	0.06
	Other crops	0.318	3.853	< 0.001 ^***^	1.00
	Woodlots	0.134	1.726	0.084	0.43
1.0	Intercept	1.511	41.921	< 0.001 ^***^	
	Cotton	−0.209	2.468	0.014 ^*^	0.88
	Maize	0.192	1.774	0.076	0.40
	Other crops	0.252	3.105	0.002 ^**^	1.00
	Woodlots	0.227	2.111	0.035^*^	0.86
1.5	Intercept	1.511	32.500	< 0.001 ^***^	
	Cotton	−0.325	2.926	0.003 ^**^	0.96
	Dwellings	0.100	1.029	0.303	0.11
	Maize	−0.037	0.349	0.727	0.05
	Other crops	0.172	1.247	0.212	0.24
	Woodlots	0.151	1.536	0.125	0.33
2.0	Intercept	1.511	28.997	< 0.001 ^***^	
	Cotton	−0.311	2.587	0.010 ^**^	0.86
	Dwellings	0.067	0.593	0.553	0.08
	Maize	−0.002	0.014	0.989	0.06
	Other crops	0.178	1.193	0.233	0.19
	Woodlots	0.187	1.318	0.187	0.23

**Table 3:** List of models that were included in the top model set (ΔAICc < 4.0) for model averaging procedure, to infer landscape effects on the abundance of *Apolygus lucorum* in cotton fields in August. Best models at each scale are indicated in bold.

Scales (km)	Model	K	logLik	AICc	ΔAICc	Weight	Adjusted *R*^2^
0.5	Other crops	3	−4.04	16.30	0.00	0.30	0.17
	Null	2	−5.99	16.99	0.68	0.21	0.00
	Maize	3	−5.3	18.78	2.48	0.09	0.02
	Dwellings	3	−5.32	18.82	2.52	0.09	0.02
	Dwellings + Other crops	4	−3.74	19.48	3.18	0.06	0.14
	Maize + Other crops	4	−3.81	19.62	3.31	0.06	0.13
	Cotton	3	−5.77	19.72	3.42	0.05	−0.05
	Cotton + Other crops	4	−3.93	19.86	3.55	0.05	0.11
	Woodlots	3	−5.89	19.96	3.66	0.05	−0.06
	Other crops + Woodlots	4	−4.05	20.10	3.79	0.04	0.10
1.0	Other crops	3	−3.12	14.42	0.00	0.29	0.27
	Maize + Other crops	4	−1.65	15.31	0.89	0.19	0.35
	Maize	3	−.19	16.56	2.15	0.10	0.15
	Null	2	−5.99	16.99	2.57	0.08	0.00
	Cotton + Other crops	4	−2.61	17.22	2.80	0.07	0.26
	Cotton	3	−4.66	17.50	3.08	0.06	0.10
	Maize + Woodlots	4	−2.79	17.57	3.16	0.06	0.24
	Maize + Other crops + Woodlots	5	−0.46	17.58	3.16	0.06	0.39
	Dwellings + Other crops	4	−3.11	18.23	3.81	0.04	0.21
	Other crops + Woodlots	4	−3.12	18.23	3.81	0.04	0.21
1.5	Maize + Other crops	4	−0.48	12.97	0.00	0.19	0.44
	Other crops	3	−2.4	12.99	0.02	0.19	0.33
	Maize + Other crops + Woodlots	5	1.6	13.46	0.49	0.15	0.54
	Maize + Woodlots	4	−0.9	13.80	0.83	0.13	0.41
	Cotton	3	−3.12	14.42	1.46	0.09	0.27
	Dwellings + Maize + Other crops	5	0.81	15.04	2.07	0.07	0.49
	Cotton + Other crops	4	−1.72	15.44	2.47	0.06	0.34
	Other crops + Woodlots	4	−2.31	16.61	3.64	0.03	0.29
	Dwellings + Maize + Woodlots	5	0.02	16.63	3.67	0.03	0.43
	Cotton + Maize	4	−2.33	16.65	3.69	0.03	0.28
	Dwellings + Other crops	4	−2.4	16.80	3.84	0.03	0.28
2.0	Maize + Other crops + Woodlots	5	3.4	9.87	0.00	0.34	0.64
	Maize + Other crops	4	1.05	9.90	0.03	0.34	0.54
	Other crops	3	−1.45	11.08	1.20	0.19	0.41
	Maize + Woodlots	4	−0.29	12.59	2.71	0.09	0.45
	Dwellings + Maize + Other crops	5	1.4	13.86	3.99	0.05	0.53

**Table 4:** Results of model averaging procedure to estimate landscape effects on abundance of *Apolygus lucorum* in cotton fields in August. Significant at: ^*^ *p* < 0.05; ^**^ *p* < 0.01; ^***^ *p* < 0.001.

Scales (km)	Variable	Estimate	z Value	Pr (>|z|)	Relative Importance
0.5	Intercept	1.548	15.126	< 0.001 ^***^	
	Cotton	−0.108	0.494	0.622	0.10
	Dwellings	−0.185	0.843	0.399	0.15
	Maize	0.184	0.824	0.410	0.14
	Other crops	0.346	1.674	0.094	0.51
	Woodlots	−0.059	0.264	0.792	0.09
1.0	Intercept	1.548	16.624	< 0.001 ^***^	
	Cotton	−0.231	1.063	0.288	0.13
	Dwellings	−0.016	0.077	0.939	0.04
	Maize	0.360	1.521	0.128	0.41
	Other crops	0.391	2.025	0.043 ^*^	0.70
	Woodlots	0.234	0.847	0.397	0.16
1.5	Intercept	1.548	18.607	< 0.001 ^***^	
	Cotton	−0.349	1.575	0.115	0.18
	Dwellings	0.198	0.860	0.390	0.13
	Maize	0.405	1.864	0.062	0.60
	Other crops	0.407	2.149	0.032 ^*^	0.72
	Woodlots	0.369	1.604	0.109	0.34
2.0	Intercept	1.548	21.308	< 0.001 ^***^	
	Dwellings	0.123	0.650	0.516	0.05
	Maize	0.345	2.186	0.029 ^*^	0.81
	Other crops	0.452	2.643	0.008 ^**^	0.91
	Woodlots	0.332	1.850	0.069	0.43

## Supplementary Materials

The following are available online at https://www.mdpi.com/2075-4450/11/3/185/s1, Figure S1: Percentage of different land cover types around sampled cotton fields within four different scales (0.5, 1.0, 1.5 and 2.0 km), Table S1: Coefficients of Spearman correlation among landscape variables at each scale. Significant at: * *p* < 0.05; ** *p* < 0.01.
