# Supplementary material for: Landscape Effects on the Abundance of Apolygus lucorum in Cotton Fields
Source: Insects. 2020 Mar 14;11(3):185. doi: 10.3390/insects11030185 (PMC7143888; doi:10.3390/insects11030185)
Supplement: Supplementary file 1 [file insects-11-00185-s001.pdf]

Supplementary materials

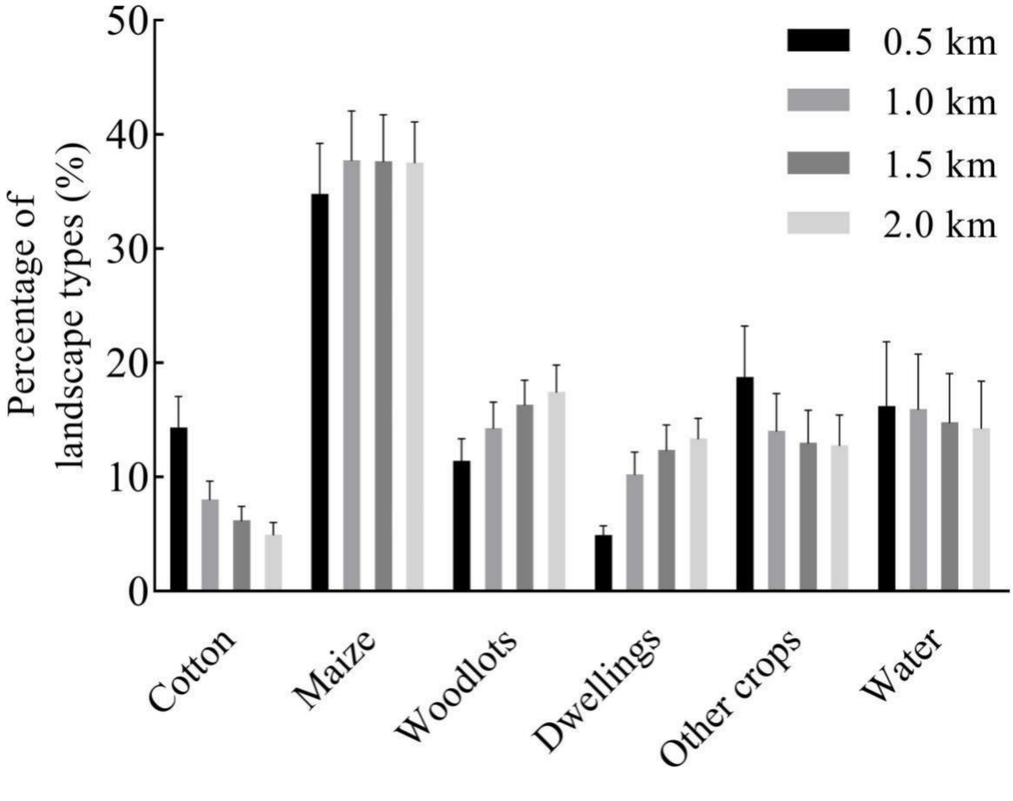

Figure S1. Percentage of different land cover types around sampled cotton fields within four different scales (0.5, 1.0, 1.5, and 2.0 km).

Table S1. Coefficients of Spearman correlation among landscape variables at each scale. Significant at: * $P < 0.05$; ** $P < 0.01$.

Scales (km)	Variables	Cotton	Maize	Woodlots	Dwellings	Other crops	Water
**0.5**	**Cotton**	1.00					
	**Maize**	−0.39	1.00				
	**Woodlots**	−0.03	−0.45	1.00			
	**Dwellings**	0.17	−0.44	0.43	1.00		
	**Other crops**	−0.24	0.18	0.00	−0.18	1.00	
	**Water**	−0.02	−0.27	−0.15	0.09	−0.70**	1.00
**1.0**		**Cotton**	**Maize**	**Woodlots**	**Dwellings**	**Other crops**	**Water**
	**Cotton**	1.00					
	**Maize**	−0.40	1.00				
	**Woodlots**	−0.03	−0.39	1.00			
	**Dwellings**	−0.06	−0.37	0.42	1.00		
	**Other crops**	−0.36	0.09	0.19	0.07	1.00	
	**Water**	0.15	−0.32	−0.19	−0.34	−0.69**	1.00
**1.5**		**Cotton**	**Maize**	**Woodlots**	**Dwellings**	**Other crops**	**Water**
	**Cotton**	1.00					
	**Maize**	−0.45	1.00				
	**Woodlots**	−0.39	−0.29	1.00			
	**Dwellings**	−0.13	−0.21	0.40	1.00		
	**Other crops**	−0.56*	0.07	0.35	0.16	1.00	

	**Water**	0.45	−0.21	−0.36	−0.47	−0.75**	1.00
**2.0**		**Cotton**	**Maize**	**Woodlots**	**Dwellings**	**Other crops**	**Water**
	**Cotton**	1.00					
	**Maize**	−0.29	1.00				
	**Woodlots**	−0.68**	−0.23	1.00			
	**Dwellings**	−0.36	−0.31	0.51*	1.00		
	**Other crops**	−0.61*	−0.03	0.54*	0.31	1.00	
	**Water**	0.62**	−0.20	−0.55*	−0.31	−0.76**	1.00
